# Robust Fine-Grained Pest Classification via Boundary-Aware Attention and Growth-Stage Supervision

**DOI:** 10.3390/insects17040423

**Published:** 2026-04-15

**Authors:** Xinliang Liu, Ruiming Zhu, Yuying Cao

**Affiliations:** 1 College of Electrical Engineering and Information, Northeast Agricultural University, Harbin 150006, China; liuxl@neau.edu.cn (X.L.);; 2 College of Medicine and Biological Information Engineering, Northeastern University, Shenyang 110169, China

**Keywords:** fine-grained pest classification, deep learning, boundary-aware attention, auxiliary label supervision

## Abstract

Accurate pest identification plays a critical role in effective pest management and sustainable agricultural production. However, fine-grained pest classification is inherently challenging due to low inter-class separability, significant intra-class variability, and complex environmental interference. To address these challenges, we develop a boundary-aware channel-spatial attention network to strengthen discriminative feature learning while suppressing background noise. The proposed attention module enhances fine-grained structural and boundary cues to improve inter-class separability under cluttered field conditions. Furthermore, auxiliary label supervision based on pest growth stages is incorporated to model developmental variations and enhance intra-class consistency. Experiments on the IP102 dataset demonstrate that the proposed method consistently outperforms state-of-the-art baselines in classification accuracy, validating its effectiveness for fine-grained agricultural pest classification. These results highlight the potential of integrating boundary-aware attention mechanisms with growth-stage supervision for robust real-world pest classification.

## Simple Summary

Accurate pest identification is essential for effective pest control and sustainable agricultural production. However, pest classification remains challenging due to subtle differences between species, large variations within the same species, and complex field environments. In this study, we develop a boundary-aware attention-based model to enhance the extraction of discriminative features while reducing background interference. Additionally, auxiliary labels are incorporated during training to improve the model’s robustness to intra-class variations. Experiments on the IP102 dataset demonstrate that the proposed approach achieves superior classification performance, highlighting its potential for practical agricultural applications.

## 1. Introduction

The rapid growth of the global population poses a significant challenge to maintaining stable and sufficient crop production [1]. Insect pests are widely recognized as a significant threat to global food security [2,3]. They cause a decrease in crop yield or even complete crop failure by nibbling on plant leaves, sucking on plant sap, and destroying root systems [4]. Additionally, the proliferation and activities of pests can facilitate the transmission of diseases, further exacerbating ecological issues within agricultural fields [5]. According to the Food and Agriculture Organization, pests are responsible for approximately 40% of global crop production losses annually [6].

Currently, several methods are employed for agricultural pest management, including chemical, biological, and physical controls. Chemical control is quick and easy to implement; however, their prolonged use may lead to increased pest resistance and environmental pollution. In contrast, biological and physical controls are more environmentally friendly, but their practical application remains constrained by strict usage requirements and slower action rates [7]. Therefore, improving early pest detection and accurate classification is essential for enabling timely interventions, reducing reliance on chemical pesticides, and ultimately enhancing crop yield and quality [8,9,10].

Traditionally, pest identification and classification have relied heavily on experienced farmers and experts. Manual pest identification is time-consuming, labor-intensive, and prone to human error due to the subtle differences in pest morphology and texture [6]. Moreover, its subjective nature and limited scalability further restrict its efficiency and reliability, particularly in large-scale agricultural monitoring scenarios [11,12,13,14,15]. As artificial intelligence and big data technologies continue to advance in agriculture, an increasing amount of research is focusing on the use of machine learning for the automatic identification of crop pests and diseases.

In the early applications of machine learning for insect identification, methods largely relied on feature extraction and traditional classifiers. Guo et al. applied a Bayesian approach to identify four types of crop infections—downy mildew, anthracnose, powdery mildew, and gray mold—based on texture and color characteristics, achieving an average accuracy of 88.48% [16]. Zhang et al. used K-means clustering to distinguish between infected and healthy regions on cucumber leaves, isolating the areas affected by disease [17]. However, these approaches had several limitations. First, many insect identification guides focused only on specific castes, such as worker ants, making it difficult to identify other castes like queens or males. Similarly, species identification for certain parasitoid species, especially those used in biocontrol programs, often depended on subtle differences in male genitalia, making it nearly impossible to accurately identify females at the species level using traditional morphological techniques [18]. Furthermore, these methods were often tailored for specific pest types, which limited their general applicability. As a result, they performed poorly in dynamic, real-world environments.

With ongoing advancements in computer vision, deep convolutional neural networks (CNNs) have demonstrated significant success across various computer vision tasks [19,20,21,22,23,24,25]. In particular, deep CNNs have achieved remarkable progress in crop disease classification. For instance, Lu et al. employed a deep multi-instance learning approach to develop an automated wheat disease diagnosis system, achieving over 95% accuracy [26]. Comprehensive grouped differentiated residual utilized a multi-branch structure of comprehensive grouped differentiation residuals to capture diverse features of tomato leaf disease across multiple dimensions and sensory fields [27]. Deng et al. proposed the multi-scale convolution module for segmenting diseased areas on tomato leaves [28]. Hasan et al. presented a compact CNN architecture that, despite its smaller scale, delivered promising results for rice leaf disease prediction, balancing accuracy with reduced time complexity [29]. However, CNNs, while effective in capturing local features, are constrained in modeling long-range dependencies, which hinders their robustness in complex pest classification tasks.

Recent studies have incorporated vision Transformers (ViTs) into pest classification tasks to address the limitations of CNNs, leveraging their self-attention mechanism to capture global contextual information [30,31]. Zhou et al. introduced a residual distillation transformer architecture that achieved 92% accuracy in classifying four types of rice leaf diseases: white leaf blight, brown spot, rice blight, and brown fly [32]. The self-attention feature fusion model demonstrated high accuracy in detecting pests in rice crops [33]. With the use of a self-attention mechanism, the spatial feature fusion and contrast-enhanced vision transformer takes three images as input, which strengthens the model’s ability to distinguish pests from noise [34].

**Figure 1:**
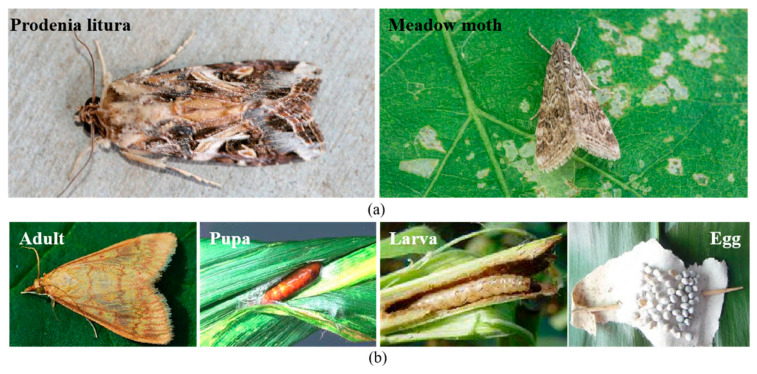
Species with similar appearances and variations in insect morphology across different development stages. (**a**) Comparison of insects with similar external appearances but belonging to different species. (**b**) Morphological variations of Corn borer across different life stages, highlighting the differences between adult, pupa, larva, and egg.

While artificial intelligence offers a potential solution to taxonomic challenges, using deep learning to classify insect pests presents several obstacles [35]. (1) Low differentiation and high variability: There is limited distinction between species, while significant variability exists within a single species, particularly across different life stages (e.g., adult, pupa, larva, and egg) [36]. As shown in Figure 1, Figure 1a compares the *Prodenia litura* and Meadow moth, two species that look strikingly similar externally. Figure 1b illustrates the morphological differences between different life stages of the Corn borer, which further complicates the task of accurate pest classification. (2) Environmental interference: Pest images in field conditions are often affected by factors such as uneven lighting, background color similarity, and partial occlusion, requiring methods to suppress these interference features [1]. (3) Data imbalance: Agricultural pest datasets often suffer from class imbalance due to data collection complexities and sample constraints. Models tend to perform well on dominant categories with larger sample sizes, but struggle with marginal categories, affecting overall recognition performance and robustness [37,38,39].

To address the above challenges, we propose a boundary-aware channel-spatial attention with growth-stage supervision network (BCSA-GSNet). BCSA-GSNet integrates a boundary-aware channel-spatial attention and growth-stage auxiliary supervision to improve discriminability and robustness. The main contributions are summarized as follows:

1. We present BCSA-GSNet, which combines (i) a boundary-aware channel-spatial attention (BCSA), (ii) growth-stage auxiliary supervision, and (iii) an imbalance-aware combined loss, achieving superior performance over strong baselines on the IP102 dataset [40].
2. We design BCSA to explicitly exploit edge cues (e.g., extracted via the Sobel operator) and dual attention to enhance boundary features while suppressing irrelevant background responses, thereby improving inter-class discrimination under cluttered field conditions.
3. We incorporate growth-stage auxiliary labels as an additional supervision signal during training to model developmental appearance changes and enhance intra-class consistency, mitigating misclassification caused by large growth-stage variations within the same species.

## 2. Materials and Methods

This section presents the overall architecture of the proposed BCSA-GSNet. The framework is built upon a pre-trained lightweight VoVNet backbone for feature extraction and incorporates multiple BCSA modules to refine discriminative representations. In addition, auxiliary supervision based on pest growth-stage annotations is incorporated during training to model intra-class developmental variations. To further address data imbalance, a combined loss function is employed to improve robustness across categories with limited samples. The detailed design of each component is described in the following subsections.

**Figure 2:**
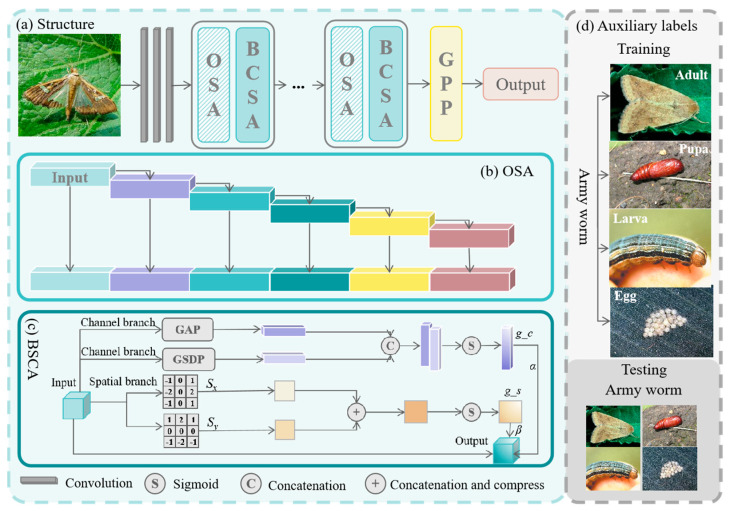
Overview of the proposed BCSA-GSNet: (**a**) Structure of the model. (**b**) Operations in the OSA block. (**c**) Architecture of the BCSA module. (**d**) Example images with auxiliary labels for training and testing, covering various stages of the Army worm (Adult, Pupa, Larva, Egg).

The overall framework of BCSA-GSNet is shown in Figure 2. First, input images are resized to a uniform size of 224 × 224 pixels. As shown in Figure 2a, the model processes the images through several convolutional layers to extract low-level features. Subsequently, the feature maps are passed through multiple one-shot aggregation (OSA) and BCSA modules. The OSA module provides multi-scale receptive field representations of various features, while the BCSA focuses on learning fine-grained details across channels and edge-specific information. Following these modules, the feature maps are processed using a generalized p-norm pooling (*GPP*) operation, adaptively focusing on high-contrast areas or subtle details. To mitigate the substantial appearance variations across different life stages of the same species, auxiliary growth-stage labels are incorporated as an additional supervision signal during training to enhance intra-class feature consistency. Notably, these auxiliary labels are only used during training and are not required during inference, thereby preserving generalization capability and practical applicability.

### 2.1. The Backbone Network

To extract discriminative representations for fine-grained pest classification, we adopt VoVNet-57 as the backbone network. Unlike standard residual networks, VoVNet leverages the OSA module, which aggregates features with varying receptive fields at the final layer of each stage. This mechanism enables the network to capture a diverse range of visual cues, from local textures to global shapes, which are crucial for distinguishing pest species that exhibit subtle inter-class differences. Moreover, VoVNet offers a superior trade-off between accuracy and inference efficiency compared to DenseNet, making it highly suitable for deployment in resource-constrained agricultural environments.

Regarding the network initialization, we employ a transfer learning strategy by loading weights pre-trained on ImageNet. Although there is a domain gap between natural scenes and agricultural images, pre-training provides a robust initialization of low-level filters (e.g., edges and patterns). This strategy significantly accelerates convergence and enhances the model’s ability to generalize on the target pest dataset, preventing overfitting that often occurs when training deep networks on limited domain-specific data from scratch.

As shown in Figure 2b, the OSA module comprises several sequential convolutional layers whose outputs are concatenated in a one-shot manner to form an aggregated feature representation. Let *f* denote the input feature map. The output of the *i*-th convolutional layer is defined as:

*y_i_* = *σ*(Conv*_i_*(*f*)), for *i* = 1, 2, …, 5, (1)

where Conv*_i_* represents the convolution operation at the *i*-th layer, and *σ* denotes the nonlinear activation function. After generating the intermediate feature maps {*y*_1_, *y*_2_, *y*_3_, *y*_4_, *y*_5_}, the OSA module aggregates them in a one-shot manner:

*y*_concat_ = concat(*y*_1_, *y*_2_, *y*_3_, *y*_4_, *y*_5_). (2)

### 2.2. Boundary-Aware Channel-Spatial Attention (BCSA)

To enhance discriminative feature learning under fine-grained and cluttered conditions, we design a BCSA module, as illustrated in Figure 2c. The module consists of a channel-attention branch and a spatial-attention branch, which capture global channel-wise statistics and spatial saliency, respectively.

Channel branch: Given an input feature map *f*, we compute its channel-wise statistical descriptors by applying global average pooling (GAP) and global standard deviation pooling (GSDP) along the spatial dimensions, denoted as *μ*(*f*) and *σ*(*f*), respectively. While *μ*(*f*) reflects the overall activation strength of each channel, *σ*(*f*) reflects the intra-channel variability across spatial locations, providing complementary second-order statistics that help characterize spatial response fluctuations. The concatenated descriptor [*μ*(*f*), *σ*(*f*)] is transformed by a learnable convolutional layer *W_c_* followed by a sigmoid activation to generate channel attention weights:

*g*_*c* = *sigmoid*(*W_c_* · [*μ*(*f*), *σ*(*f*)]). (3)

Spatial branch: To explicitly emphasize structural and boundary information, the input feature map *f* in the spatial branch is processed by two fixed Sobel kernels, *S_x_* and *S_y_*, to extract the horizontal and vertical edge responses, respectively:

(4) $$\begin{array}{l} E_{x}=S_{x}*f\\ E_{y}=S_{y}*f \end{array}$$

where *S_x_* and *S_y_* denote the standard fixed Sobel kernels in the horizontal and vertical directions, respectively, and * denoted convolution. The two edge responses are then fused by element-wise addition and refined through a learnable convolution *W_s_*, followed by a sigmoid activation, to generate the spatial attention weights:

*g*_*s* = *sigmoid*(*W_s_* · (*E_x_* + *E_y_*)). (5)

**Feature refinement**: The final output is obtained through residual-style multiplicative modulation:

*output* = *f* × (1 + *α* · *g*_*c*) × (1 + *β* · *g*_*s*), (6)

where *α* and *β* are hyperparameters controlling the relative contributions of channel and spatial attention. The residual formulation preserves the original feature information while adaptively enhancing discriminative responses.

### 2.3. Generalized p-Norm Pooling (GPP)

In fine-grained pest classification, discriminative cues often reside in localized high-response regions, while background noise may dilute these signals. Conventional GAP treats all spatial locations equally, potentially weakening salient activations. To address this limitation, we adopt *GPP*, defined as:

(7) $$GPP(x)={\left(\frac{1}{H\cdot W}\sum_{h=1}^{H}\sum_{w=1}^{W}x_{h,w}^{p}\right)}^{\frac{1}{p}},$$

where *H* and *W* are the height and width of the feature map, respectively. *p* is a learnable parameter controlling the sharpness of the pooling response.

By introducing an exponent parameter *p*, *GPP* generalizes mean pooling and enables a smooth transition between mean-like and max-like behaviors. When *p* = 1, *GPP* reduces to GAP. For *p* > 1, the pooling operation becomes increasingly sensitive to high activation values, thereby emphasizing strong feature responses. Conversely, smaller values of *p* produce smoother aggregation behavior by reducing the dominance of extreme responses. By learning *p*, the model can adaptively adjust the pooling behavior according to the feature distribution.

### 2.4. Loss Function

Cross-entropy loss has been widely used in classification tasks [41]. To balance training stability and address class imbalance, we further combine it with focal loss [42] and propose a mixed loss function.

(8) $$L_{\mathrm{Total}}=\lambda \cdot L_{\mathrm{CE}}+(1-\lambda )\cdot L_{\mathrm{FL}},$$

where *L*_CE_ and *L*_FL_ are the cross-entropy loss and the focal loss, respectively. *λ* controls the relative importance of the two losses.

In the early training phases, the model relies more on the cross-entropy loss for stable learning, and as training progresses, the focus shifts toward harder samples by increasing the contribution of the focal loss. To achieve this dynamic adjustment, *λ* is updated as follows:

(9) $$\lambda =\lambda_{\max}\cdot (1-\frac{t}{T}),$$

where *t* is the current epoch, *T* is the total number of training epochs, and *λ*_max_ is the initial value of *λ*. As the number of training epochs increases, *λ* decreases, giving more importance to the focal loss.

### 2.5. Auxiliary Label Supervision

**Figure 3:**
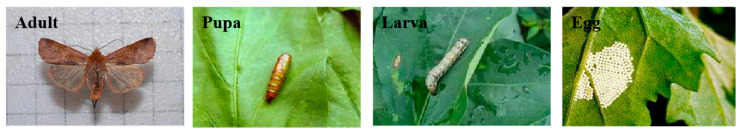
Images of the Beet army worm at different growth stages: adult, pupa, larva, and egg. These stages exhibit significant morphological differences, demonstrating the intra-class variability that complicates classification.

In fine-grained agricultural pest classification, significant intra-class variability often arises from substantial appearance changes across different growth stages of the same species. As shown in Figure 3, the Beet army worm, despite being classified under a single label, shows marked differences across its growth stages: Adult, Pupa, Larva, and Egg. The adult stage appears as a moth, the pupa is a chrysalis, the larva is a worm-like caterpillar, and the egg is a cluster of small white discs on leaves. Because the same label covers visually unrelated forms, the supervision signal becomes inconsistent: the network is forced to associate moth-like, chrysalis-like, and worm-like patterns with a single category, which enlarges intra-class variance and weakens the compactness of class-specific representations. This, in turn, blurs the decision boundary between categories and increases the risk of confusion with other species that resemble a particular growth stage.

**Figure 4:**
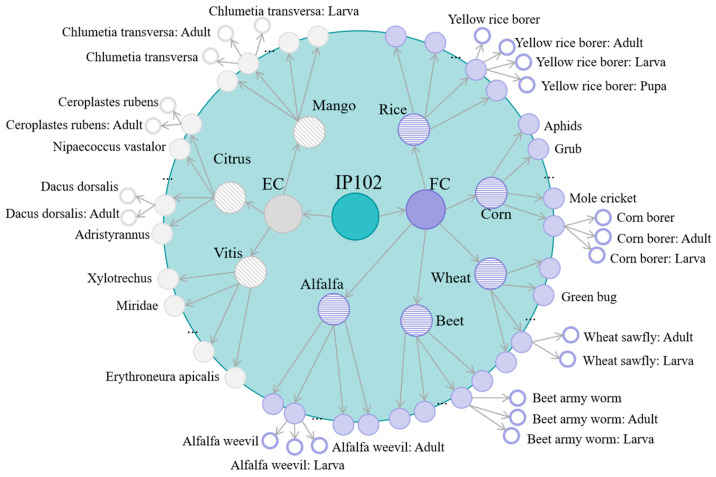
Hierarchical taxonomy of the IP102 dataset and the assignment of auxiliary growth-stage labels. For clarity, only a subset of the species-level nodes is displayed. Species whose samples can be further divided into distinct developmental stages and whose stage-specific subgroup size exceeds the predefined threshold are expanded into stage-aware subclasses (illustrated by the peripheral branch nodes), such as Yellow rice borer and similar eligible categories. In contrast, species with insufficient stage-specific samples remain under their original labels.

To alleviate the above issue, we introduce auxiliary supervision based on growth-stage annotations during training. The growth stages are categorized into four types: Adult, Larva, Pupa, and Egg. For species where sufficient samples are available for a particular stage, stage-specific auxiliary labels are introduced in addition to the original species label. In implementation, considering that the open-source IP102 dataset is highly imbalanced, some growth-stage-specific groups contain only a limited number of samples. If such small groups were directly split into new classes, the model would be unable to learn robust features for them and the performance might even degrade. Therefore, a new growth-stage-specific label is assigned only when the corresponding subgroup contains more than 30 images. Following this strategy, the training labels are refined from the original 102 classes to 168 classes. Figure 4 illustrates the hierarchical organization of the IP102 dataset and the integration of auxiliary growth-stage labels.

During training, the model is optimized to classify these 168 refined classes, enabling it to learn more discriminative and growth-stage-aware representations. During testing, however, the predictions of all growth-stage-specific subclasses are mapped back to their corresponding original pest categories, and the final evaluation is still performed on the original 102 classes.

## 3. Results

### 3.1. Experimental Settings

The experiments are conducted using an Intel Core i7-12700F CPU and an NVIDIA GeForce RTX 3060 GPU. The implementation is carried out with PyTorch 1.10.0. The backbone network is VoVNet-57, with an input resolution of 224 × 224, a batch size of 32, and a maximum of 500 training epochs. We use the AdamW optimizer with an initial learning rate of 1 × 10^−3^, *β*_1_ = 0.9, *β*_2_ = 0.999, and a weight decay of 5 × 10^−4^. In addition, label smoothing of 0.1 is applied, and early stopping with a patience of 30 epochs is adopted. To avoid sharp fluctuations and promote smoother convergence, a warm-up learning-rate strategy is employed during the initial phase of training, with the parameter settings following the default configuration.

For data preprocessing, images are normalized using the ImageNet mean and standard deviation for each channel. During training, images are first resized to 1.1 × the target input size, randomly cropped to the final resolution, and then normalized using ImageNet statistics. During evaluation, images are resized, center-cropped, and normalized using the same ImageNet statistics. Regarding the dataset protocol, we use the publicly available IP102 dataset and directly follow its official train/validation/test split, which avoids ambiguity caused by customized partitioning and enables fairer comparison with previous methods evaluated on the same benchmark.

### 3.2. Evaluation Indicators

The evaluation of classification performance is based on several comprehensive metrics, including accuracy, F1-score, macro F1-score (macro F1), and weighted F1-score (weighted F1). Accuracy is the most straightforward metric, representing the overall performance of the classifier. It is calculated as the ratio of correctly classified samples to the total number of samples, expressed as:

(10) $$Accuracy=\frac{T_{right}}{T_{all}},$$

where *T_right_* denotes the number of correct classifications and *T_all_* is the total number of samples.

The F1-score is the harmonic mean of precision and recall:

(11) F1−scorei=2⋅Precisioni⋅RecalliPrecisioni+Recalli,

(12) $$\mathrm{Pr}ecision_{i}=\frac{TP_{i}}{TP_{i}+FP_{i}},$$

(13) Recalli=TPiTPi+FNi,

where *TP_i_*, *FP_i_*, and *FN_i_* denote the numbers of true positives, false positives, and false negatives for class *i*, respectively.

Considering that class imbalance is common in fine-grained agricultural pest datasets, two variants of the F1-score—macro F1 and weighted F1—are adopted to provide a more comprehensive evaluation of classification performance. Macro F1 is the unweighted average of the F1-scores across all classes, treating each class equally regardless of its sample size. It is computed as follows:

(14) $$\mathrm{macro}\;F1=\frac{1}{n}\sum_{i=1}^{n}F1-{\mathrm{score}}_{i},$$

where *n* is the number of classes.

In contrast, the weighted F1 accounts for class distribution by computing the F1-score for each class and then taking the weighted average based on the number of samples in each class:

(15) $$\mathrm{weighted}\;F1=\frac{\sum_{i=1}^{n}num_{i}\cdot F1-{\mathrm{score}}_{i}}{\sum_{i=1}^{n}num_{i}}$$

where *num_i_* represents the number of samples in class *i*.

### 3.3. Experimental Results

#### 3.3.1. Classification Results

**Figure 5:**
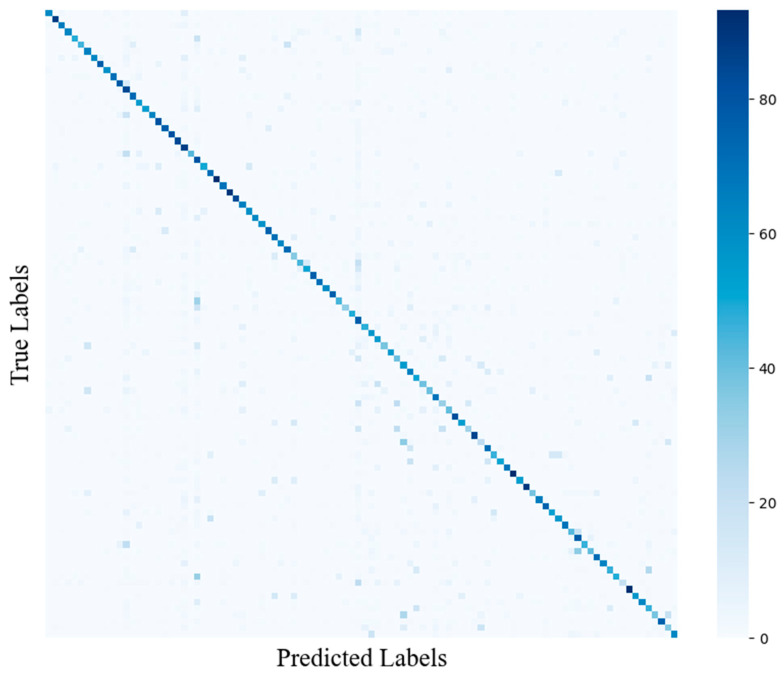
Confusion matrix of the model on the IP102 dataset. In the matrix, color intensity reflects the frequency of predictions for each true-predicted class pair, with darker values indicating higher correspondence. The diagonal elements represent correctly classified samples.

Figure 5 presents the confusion matrix of the proposed model on the IP102 dataset. Due to the large number of categories (102 classes), numeric indices are used for clarity, and the mapping between class IDs and category names is provided in Table 1. As shown in Figure 5, the diagonal entries exhibit higher intensity, indicating that the model achieves strong per-class recognition performance for the majority of categories. Nevertheless, sparse off-diagonal activations reveal remaining confusions between certain class pairs. For example, for *Aphis citricola* Vander Goot (class ID 4), 63.21% of samples are correctly classified, whereas 13.21% are misclassified as Aphids (class ID 53). Both categories correspond to aphids with highly similar morphology and small-scale appearance under cluttered leaf backgrounds, making them difficult to distinguish reliably.

#### 3.3.2. Comparison with Other State-of-the-Art Classification Models

Table 2 presents the comparison results of BCSA-GSNet and several fine-grained image classification methods on the IP102 dataset. To assess the stability and reliability of the proposed method, we conduct three independent runs and report the mean, standard deviation, and 95% confidence interval (CI) of the results. Across the three runs, our method achieves 68.87 ± 0.83 Accuracy (95% CI: [66.82, 70.92]), 62.29 ± 0.63 Macro-F1 (95% CI: [60.72, 63.86]), and 68.54 ± 0.74 Weighted-F1 (95% CI: [66.70, 70.38]), outperforming all competing methods. These results show that the proposed method not only has superior classification capability but also maintains stable and robust performance across multiple runs under class-imbalanced conditions.

We further conduct a paired statistical significance test on the accuracy results using McNemar’s exact test. Since the reported accuracy is the mean over three independent runs, while McNemar’s test requires sample-wise predictions from a single run, we used the median-performing run of our method for comparison with the strongest baseline (SFCE-VT). The McNemar test yields *p* < 0.05, indicating that the accuracy improvement of our method over the strongest baseline is statistically significant.

Regarding model complexity, BCSA-GSNet contains 35.65 M parameters. Although GhostNet and MnasNet are much lighter, their performance is significantly inferior. Compared with DNVT, which has a comparable parameter size (35.40 M), BCSA-GSNet achieves notably better results on all three metrics. Meanwhile, larger models such as DPN, ViT, and SFCE-VT still do not outperform the proposed method, which further demonstrates that BCSA-GSNet achieves a more favorable balance between classification performance and model complexity.

**Figure 6:**
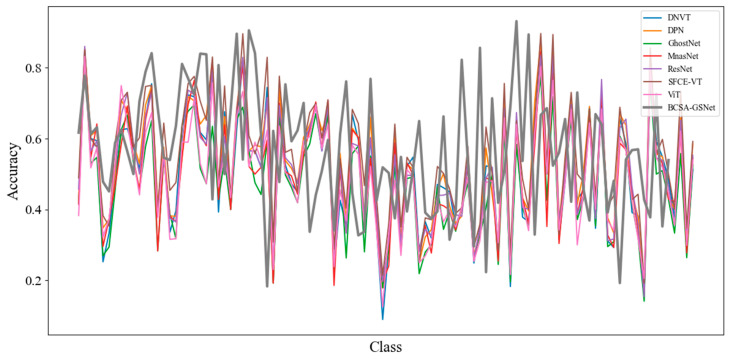
Class-wise accuracy comparison of different methods on the IP102 dataset, with our method highlighted in bold gray line. The correspondence between class IDs and labels is provided in Table 1.

Additionally, Figure 6 compares the per-class accuracy of different methods for the IP102 dataset, where each curve corresponds to a method. Overall, our method achieves the highest per-class accuracy in 78 out of 102 classes (76.47%), demonstrating a clear advantage in terms of category-wise performance. Additionally, Figure 6 compares the per-class accuracy of different methods on the IP102 dataset, where each curve corresponds to a method. Overall, our method achieves the highest per-class accuracy in 78 out of 102 classes (76.47%), indicating a clear advantage in category-wise performance. Although there are classes in which other methods obtain higher accuracy, such variability is expected in fine-grained recognition due to intra-class variation across growth stages and class-frequency imbalance, which may favor different architectural biases on different subsets of categories. Importantly, BCSA-GSNet remains the top-performing method for the majority of classes, suggesting that the proposed boundary-aware attention and auxiliary supervision help learn more robust and discriminative representations.

#### 3.3.3. Ablation Experiment of Each Module

To validate the effectiveness of each module in the model, we conduct ablation experiments to assess the performance of individual components. The BCSA-GSNet consists of four core modules—pre-training strategy, BCSA, auxiliary label supervision, and hybrid loss function. As shown in Table 3, starting with the baseline model, we progressively introduce each module. The introduction of the pre-training strategy significantly improved the model’s performance, increasing accuracy by 8.35%. Using ImageNet pre-trained weights provides robust low-level feature extractors, improving generalization on the target dataset. The integration of the BCSA and auxiliary label supervision enhances the model’s ability to learn edge features and distinguish between different growth stages of the same species, improving classification performance in complex backgrounds and mitigating confusion caused by highly variable samples. Finally, the incorporation of the hybrid loss function helps the model achieve a final accuracy of 68.75%.

(1) Additional analysis of BCSA

To further investigate the contribution of BCSA, we conduct two additional analyses from the perspectives of environmental interference robustness and discriminative representation learning. First, to evaluate the robustness of BCSA under complex background conditions, we construct an additional subset consisting of samples with relatively cluttered backgrounds. Specifically, five images with complex background interference were selected from each category, resulting in a total of 510 samples. We then compare the performance of the models with and without BCSA on this subset. The results show that the model equipped with BCSA achieved an accuracy of 60.78%, compared with 55.88% for the model without BCSA. This result provides direct evidence that BCSA improves recognition robustness in the presence of environmental interference.

Furthermore, to analyze the effect of BCSA on discriminative feature learning, we analyze the classification results for several groups of visually similar categories. For example, in the beetle/blister beetle/weevil group, the accuracy increased from 54.28% to 67.00% after introducing BCSA, while in the planthopper/leafhopper group, the accuracy improved from 66.72% to 73.27%. These results further indicate that BCSA enhances discriminative representation learning and helps reduce confusion among visually similar categories.

(2) Additional analysis of growth-stage auxiliary supervision

To further clarify the contribution of growth-stage labels, we focus on species recognition across different developmental stages. The results indicate that 89.36% of the evaluated species benefited from growth-stage auxiliary supervision. Representative examples are provided in Table 4, where Acc w/GS denotes the species-level accuracy obtained with growth-stage auxiliary supervision, and Acc w/o GS denotes the species-level accuracy obtained without growth-stage auxiliary supervision. The accuracy of Alfalfa weevil increases from 36.08% to 47.77%, Corn borer improves from 57.84% to 69.22%, and *Phyllocnistis citrella* Stainton increases from 55.74% to 69.67%. Similar improvements are also observed for *Limacodidae*, Rice leaf roller, and Wheat blossom midge. These results further demonstrate that growth-stage auxiliary supervision helps the model learn more stable discriminative features across different growth stages.

## 4. Discussion

**Figure 7:**
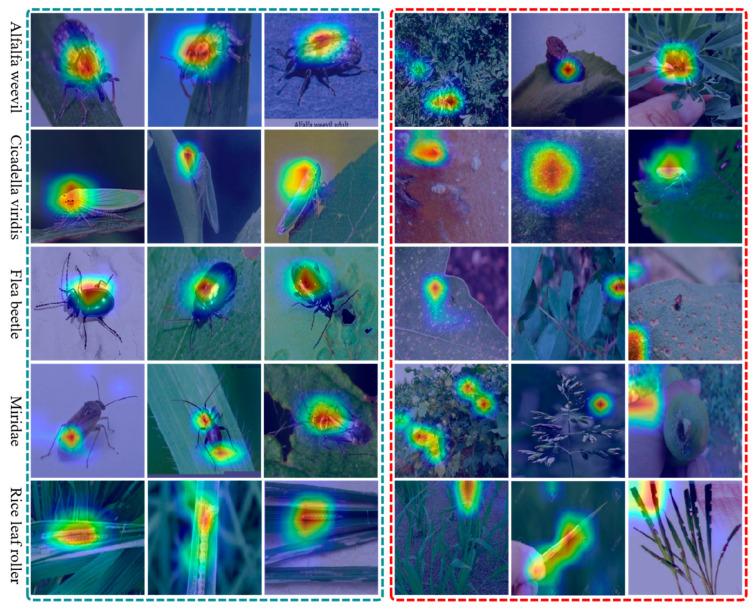
Visualization of the model performance on five insect species. Representative samples of *Alfalfa weevil*, *Cicadella viridis*, *Flea beetle*, Miridae, and Rice leaf roller are shown together with their corresponding response heatmaps. Each row corresponds to one species. Correctly classified examples are enclosed by blue dashed boxes, whereas misclassified examples are highlighted by red dashed boxes. In the heatmaps, colors ranging from blue to red indicate increasing levels of model attention, with red/yellow regions representing the most discriminative areas contributing to the final prediction.

To further understand what the proposed model focuses on during classification, we provide the visualization results in Figure 7. The model tends to attend to discriminative and biologically meaningful regions rather than relying on coarse background correlations. For the Alfalfa weevil class, the activation maps in the blue box are consistently concentrated on the insect body, especially around the head-thorax region and the main body contour, even under changes in scale, pose, illumination, and background clutter. This suggests that the network captures stable morphological cues of the adult stage instead of being distracted by leaf texture, shadows, or surrounding vegetation. Moreover, the highlighted regions are not limited to a single local response; in many cases, they extend along the object outline, indicating that the model preserves structural information and benefits from enhanced boundary perception. Such behavior is particularly important in field images, where pests often occupy only a small portion of the scene and may be partially blended with the background.

Despite the overall performance gains, misclassifications still occur in challenging scenarios. Since growth-stage labels are sparse and only partially available, supervision for rare stages remains limited, which can result in confusion among visually similar species. Furthermore, the long-tailed class distribution may bias representation learning toward majority classes, restricting the diversity of features learned for minority categories and weakening the effectiveness of boundary enhancement when data are scarce. A promising direction for future work is to incorporate data augmentation methods tailored to long-tailed and stage-imbalanced settings. In particular, augmentation schemes designed for minority classes, rare growth stages, and hard-to-distinguish samples may improve data diversity, enhance feature robustness, and further strengthen generalization in complex agricultural scenarios.

## 5. Conclusions

In this work, we addressed the challenges of fine-grained agricultural pest classification, particularly low inter-class separability, high intra-class variability, and environmental interference. In the proposed BCSA-GSNet, the BCSA module strengthens boundary and texture representations while suppressing background noise, and the auxiliary growth-stage labels enable the model to better capture developmental variations within species. Extensive experiments conducted on the IP102 dataset demonstrate that the proposed method consistently outperforms existing state-of-the-art approaches. The results validate the effectiveness of combining boundary-aware attention with stage-aware supervision for improving fine-grained recognition performance under complex agricultural conditions.

## Figures and Tables

**Table 1:** Mapping of category IDs to their corresponding labels in the IP102 dataset.

ID.	Class	ID.	Class	ID.	Class
1	Adristyrannus	35	*Prodenia litura*	69	Grain spreader thrips
2	*Aleurocanthus spiniferus*	36	*Pseudococcus comstocki* Kuwana	70	Green bug
3	*Ampelophaga*	37	*Rhytidodera bowrinii* White	71	Grub
4	*Aphis citricola* Vander Goot	38	Rice Stemfly	72	*Large cutworm*
5	*Apolygus lucorum*	39	*Salurnis marginella* Guerr	73	*Legume blister beetle*
6	*Bactrocera tsuneonis*	40	*Scirtothrips dorsalis* Hood	74	Longlegged spider mite
7	Beet spot flies	41	*Sternochetus frigidus*	75	*Lytta polita*
8	*Brevipoalpus lewisi* McGregor	42	Tetradacus c *Bactrocera minax*	76	*Meadow moth*
9	*Ceroplastes rubens*	43	Thrips	77	*Mole cricket*
10	*Chlumetia transversa*	44	*Toxoptera aurantii*	78	*Odontothrips loti*
11	*Chrysomphalus aonidum*	45	*Toxoptera citricidus*	79	*Oides decempunctata*
12	*Cicadella viridis*	46	*Trialeurodes vaporariorum*	80	*Paddy stem maggot*
13	Cicadellidae	47	*Unaspis yanonensis*	81	*Parathrene regalis*
14	*Colomerus vitis*	48	*Viteus vitifoliae*	82	*Peach borer*
15	*Dacus dorsalis*(Hendel)	49	Xylotrechus	83	*Penthaleus major*
16	*Dasineura* sp.	50	Alfalfa plant bug	84	Red spider
17	*Deporaus marginatus* Pascoe	51	Alfalfa seed chalcid	85	Rice gall midge
18	*Erythroneura apicalis*	52	*Alfalfa weevil*	86	Rice leaf caterpillar
19	*Icerya purchasi* Maskell	53	Aphids	87	Rice leaf roller
20	*Lawana imitata* Melichar	54	Army worm	88	Rice leafhopper
21	Limacodidae	55	Asiatic rice borer	89	Rice shell pest
22	Locustoidea	56	Beet army worm	90	Rice water weevil
23	*Lycorma delicatula*	57	Beet fly	91	*Sericaorient alismots* chulsky
24	Mango flat beak leafhopper	58	*Beet weevil*	92	Small brown plant hopper
25	Miridae	59	*Bird cherry-oataphid*	93	Tarnished plant bug
26	*Nipaecoccus vastalor*	60	Black cutworm	94	*Therioaphis maculata* buckton
27	*Panonchus citri* McGregor	61	*Blister beetle*	95	Wheat blossom midge
28	*Papilio xuthus*	62	*Brown plant hopper*	96	*Wheat phloeothrips*
29	*Parlatoria zizyphus* Lucus	63	Cabbage army worm	97	Wheat sawfly
30	*Phyllocnistis citrella* Stainton	64	*Cerodonta denticornis*	98	White backed plant hopper
31	*Phyllocoptes oleiverus* Ashmead	65	Corn borer	99	White margined moth
32	*Pieris canidia*	66	English grain aphid	100	Wireworm
33	*Polyphagotars onemus latus*	67	*Flax budworm*	101	Yellow cutworm
34	*Potosiabre vitarsis*	68	*Flea beetle*	102	Yellow rice borer

**Table 2:** Comparison of classification performance across different methods on IP102 dataset.

Methods	Acc	Macro F1	Weighted F1	Params (M)
DPN	59.19%	54.32%	58.68%	76.35
GhostNet	51.43%	48.71%	51.14%	3.90
MnasNet	56.34%	51.34%	55.91%	3.11
DNVT	57.56%	52.32%	57.23%	35.40
ResNet	57.04%	53.08%	56.75%	46.23
ViT	53.22%	50.06%	53.00%	86.40
SFCE-VT	62.00%	56.76%	61.72%	99.60
BCSA-GSNet	68.87%	62.29%	68.54%	35.65

**Table 3:** Ablation analysis on the components of BCSA-GSNet. √ indicates that the corresponding module is used.

Module				Acc	Macro F1	Weighted F1
Pre-Training	BCSA	Auxiliary Label	Loss			
				62.00%	56.76%	61.72%
√				67.18%	60.69%	66.91%
√	√			67.62%	61.06%	67.22%
√	√	√		67.70%	61.52%	67.33%
√	√	√	√	68.87%	62.29%	68.54%

**Table 4:** Representative species-level accuracy improvements after introducing growth-stage auxiliary supervision.

Class	Count	Acc w/o GS	Acc w/GS	Improvement
*Alfalfa weevil*	158	36.08%	47.77%	11.69%
Corn borer	510	57.84%	69.22%	11.37%
*Dacus dorsalis* (Hendel)	132	64.39%	72.52%	8.13%
Limacodidae	421	70.55%	81.71%	11.16%
*Phyllocnistis citrella* Stainton	122	55.74%	69.67%	13.93%
*Prodenia litura*	392	56.12%	65.04%	8.92%
Rice leaf roller	335	67.76%	77.74%	9.98%
Wheat blossom midge	146	85.62%	92.47%	6.85%

## Data Availability

The data presented in this study are available in IP102 dataset at https://doi.org/10.1109/CVPR.2019.00899, reference number [40]. These data were derived from the following resources available in the public domain: https://github.com/xpwu95/IP102, accessed on 13 April 2026.
